# Pathogen Identification and Pathogenicity of Fig (*Ficus carica* L.) Branch Canker Disease in Kashi, Xinjiang

**DOI:** 10.3390/jof12030164

**Published:** 2026-02-25

**Authors:** Pan Xie, Lingkai Xu, Wenwen Gao, Hongyue Li, Qian Zheng, Yuxuan Wang, Qiuyan Han, Canpeng Fu, Shuaishuai Sha

**Affiliations:** 1 School of Advanced Agricultural Sciences, Kashi University, Kashi 844008, China; 2 Research Center for Crop Biotechnology Breeding and Smart Cultivation in Southern Xinjiang, Kashi 844000, China

**Keywords:** *Alternaria alternata*, fig (*Ficus carica*), fungal pathogen, *Fusarium proliferatum*, orchards, pathogenicity

## Abstract

Little is known about the fungal pathogens responsible for fig (*Ficus carica* L.) branch canker in the Kashi region of Xinjiang, China. Using a combination of morphological characterization and multilocus sequence analyses of ITS, TEF1-α, and RPB2, we identified fungal isolates obtained from cankered fig branches collected in commercial orchards in this region. The pathogenicity of representative isolates was evaluated by artificial inoculation of fig branches under natural field conditions. Two dominant fungal species, *Fusarium proliferatum* and *Alternaria alternata*, were consistently isolated from diseased tissues. In inoculation assays, both species induced typical branch canker lesions similar to those observed in the field. Lesions caused by *F. proliferatum* were generally larger than those induced by *A. alternata*. The original pathogens were successfully re-isolated from the inoculated branches, thereby fulfilling Koch’s postulates. This study represents the first report of *F. proliferatum* and *A. alternata* as causal agents of fig branch canker in Xinjiang and expands the known spectrum of pathogens associated with fig branch diseases. These findings provide a scientific basis for improved disease monitoring and the development of sustainable management strategies in local fig orchards.

## 1. Introduction

Fig (*Ficus carica* L.), one of the most important subtropical fruit crops worldwide, is valued for its high nutritional, medicinal, and ecological benefits. In arid and semi-arid regions such as the Mediterranean basin, Central Asia, and southern Xinjiang (China), fig is a key economic species (https://www.fao.org/faostat/zh/#data/QCL, accessed on 20 November 2024). Rapid expansion of protected cultivation in southern Xinjiang has elevated fig into a pillar industry that now drives local agricultural restructuring and improves farmer incomes. However, disease—especially perennial canker and branch dieback—is increasingly constraining orchard yield stability and production efficiency.

Branch diseases typically infect lignified tissues, exhibit long incubation periods and latent infection phases, and are difficult to manage once established. Consequently, affected trees often show reduced vigor, progressive branch decline, and in severe cases, whole-tree death, leading to substantial long-term economic losses [1,2]. Globally, fig branch canker and dieback diseases have been predominantly associated with fungal pathogens, especially species belonging to the families Botryosphaeriaceae and Diaporthaceae. Among these, *Phomopsis cinerascens* (Sacc.) Sacc., *Lasiodiplodia theobromae* (Pat.) Griffon & Maubl., and *Neofusicoccum parvum* (Pennycook & Samuels) Crous, Slippers & A.J.L. Phillips, are among the most frequently reported causal agents, highlighting the importance of wood-inhabiting fungi in the etiology of fig branch diseases. These fungi typically enter host tissues through pruning wounds, sunscald cracks, or frost injuries, and cause cortex necrosis, xylem discoloration, and shoot dieback [3,4,5]. Recent multilocus-based studies have further expanded the known spectrum of pathogens, with *N. parvum* reported in Italy [6], *Neoscytalidium dimidiatum* identified in California [7], and a novel *Neocosmospora* species reported from Iran [8].

Although fungal pathogens are the predominant causal agents of fig branch canker and dieback worldwide, bacterial pathogens have also been reported to cause similar symptoms in fig trees, including bacterial canker and stem soft rot, that may lead to branch necrosis [9,10]. Mixed fungal–bacterial infections are not uncommon in woody plants under stress conditions and may exacerbate disease severity. Collectively, these findings highlight the marked regional diversity and ecological adaptation of fungal pathogens associated with fig branch diseases. However, despite the strong regional specificity and ecological differentiation observed for fig branch canker and dieback in arid and semi-arid regions of northwestern China—particularly in southern Xinjiang—systematic epidemiological investigations, pathogen isolation, and accurate identification of the causal agents remain lacking. This knowledge gap hampers our understanding of local disease patterns and limits the development of effective and sustainable disease-management strategies.

The cosmopolitan fungi *Fusarium proliferatum* and *Alternaria alternata* occur frequently in agroecosystems. *Fusarium proliferatum* is primarily soilborne and, through root penetration, blocks xylem vessels and induces wilt, making it a major causal agent of vascular wilt diseases in many herbaceous crops [11]. Some *Fusarium* species can also infect woody stems through wounds, producing localized necrosis or internal wood discoloration [12]. Their pathogenesis is associated with the secretion of cell-wall-degrading enzymes and phytotoxic metabolites such as fusaric acid that damage cambial and parenchymatous tissues. In contrast, *Alternaria* spp. have traditionally been regarded as airborne pathogens that attack leaves and fruits, commonly causing leaf spot, fruit rot, and black mold [13,14]. However, *A. alternata* has been reported to infect apple branches and induce typical canker and dieback symptoms, with pathogenicity confirmed by artificial inoculation assays [15]. These observations suggest that under conditions of environmental stress or tissue wounding, certain endophytic or saprophytic fungi may shift from a latent to an active pathogenic lifestyle, thereby causing branch canker and dieback diseases [16].

The Kashi region of Xinjiang is characterized by an arid climate, large diurnal temperature fluctuations, hot summers, and frequent winter freeze–thaw cycles. These conditions promote the formation of microcracks and sunburn injuries on fig branch surfaces, which serve as potential entry points for pathogenic fungi [17]. In recent years, the prevalence of fig branch canker manifested as longitudinal bark necrosis, sunken lesions, and gummosis has increased markedly in local orchards to the point that it has now become a major constraint to fig production. While latent pathogens commonly invade host tissues through wounds in drought- and stress-prone ecosystems [18,19], no systematic research on fig branch diseases has been performed in Xinjiang. The aim of this study was to investigate the fungal pathogens associated with canker symptoms from this region using a combined morphological and molecular approach, to further characterize their pathological features, and to verify their pathogenicity to fig.

## 2. Materials and Methods

### 2.1. Field Survey and Pathogen Isolation

Between 2023 and 2025, commercial fig orchards in the Kashi region of southern Xinjiang were surveyed. Fig trees (cv. ‘Zao-huang’), ~10–15 years old, exhibited conspicuous woody branch cankers and shoot dieback symptoms. A total of 40 symptomatic branch samples showing typical canker or blight symptoms were collected from four commercial orchards in Artush (39°42′58″ N, 76°10′06″ E) and Kashi (39°28′05″ N, 75°59′38″ E) cities, then transported to the Plant Pathology Laboratory, College of Modern Agriculture, Kashi University.

Fungal pathogens were isolated using a standard tissue-segment method [20]. With a sterile scalpel, wood–bark tissue fragments (~5 × 5 × 5 mm) were cut from the advancing margins of active lesions. Surface sterilization was performed by sequential immersion in 75% (*v*/*v*) ethanol for 30 s, 1% (*w*/*v*) sodium hypochlorite (NaOCl) for 3 min, followed by three rinses in sterile distilled water. After air-drying under sterile conditions, the tissue segments were placed onto potato dextrose agar (PDA; Solarbio, Beijing, China) and incubated at 25 °C in darkness for 5 days. Emerging fungal colonies with distinct morphological characteristics were subcultured by transferring hyphal tips onto fresh PDA plates to obtain pure cultures. Purified isolates were incubated on PDA at 25 °C for 5 days and subsequently stored at 4 °C for further morphological, molecular, and pathogenicity analyses.

### 2.2. Pathogen Morphology

To characterize the morphology of fungal isolates, 5 mm-diameter mycelial plugs taken from the actively growing margins of 3-day-old pure cultures were placed on PDA and incubated at 25 °C for 5–10 days under a 14/10 h light/dark photoperiod. Colony expansion was recorded daily, and the radial growth rate (mm day^−1^) was calculated from the mean of two perpendicular colony diameters measured during the first 3 days of incubation. Colony characteristics, including color, texture, zonation, aerial mycelium development, and exudate production, were recorded on days 5 and 10.

To induce sporulation, a sequential light–dark regime was applied: mycelial plugs were initially incubated in complete darkness at 25 °C for 4 days, followed by exposure to near-UV light under a 12/12 h light/dark cycle for an additional 6–7 days. After 7–10 days of total incubation, conidial morphology was examined using an Olympus BX43 light microscope (Olympus, Tokyo, Japan). Sporulating mycelia were gently removed from the colony margins and mounted in sterile distilled water; 30–50 conidia per isolate were measured at ×400 magnification to determine conidial shape, septation, pigmentation, and dimensions.

### 2.3. Molecular Identification

Genomic DNA was extracted from 5-day-old PDA cultures using an Ezup Column Fungal Genomic DNA Kit (Sangon Biotech, Shanghai, China). The internal transcribed spacer (ITS), translation elongation factor 1-alpha (TEF1-α), and RNA polymerase II second largest subunit (RPB2) loci were amplified using the primer pairs ITS1/ITS4 [21], EF1-728F/EF1-986R [22], and RPB2-5f2/RPB2-7cr [23], respectively. Each 25 μL PCR reaction mixture contained 1 μL of genomic DNA, 0.5 μL of each primer, 12.5 μL of 2× Taq Master Mix, and sterile double-distilled water (ddH_2_O) added to a final volume of 25 μL. PCR amplification conditions followed those described by He et al. [21]. The protocol included an initial denaturation at 94 °C for 4 min, followed by 35 cycles of denaturation at 94 °C for 30 s, annealing at 55 °C for ITS or 59 °C for TEF1-α and RPB2 for 30 s, and extension at 72 °C for 30 s for ITS or 60 s for TEF1-α and RPB2. A final extension step was performed at 72 °C for 10 min. PCR amplicons were visualized on 1.0% (*w*/*v*) agarose gels, purified using a TIANquick Midi Gel Extraction Kit (Tiangen, Beijing, China), and bidirectionally sequenced by Tsingke Biotechnology Co., Ltd. (Xi’an, China). Raw sequences were edited and assembled using BioEdit software version 7.0.0. The assembled sequences were queried against the NCBI nucleotide database using BLASTn (https://blast.ncbi.nlm.nih.gov/Blast.cgi, accessed on 11 November 2025) for preliminary taxonomic identification and were subsequently submitted to GenBank under assigned accession numbers.

### 2.4. Phylogenetic Analysis

All newly generated sequences were deposited in the NCBI GenBank database, and closely related reference sequences were retrieved for preliminary taxonomic assignment. Reference sequences of the ITS, TEF1-α, and RPB2 loci from phylogenetically related strains were downloaded from GenBank. The three loci were aligned independently using the MAFFT v7 online server (https://mafft.cbrc.jp/alignment/server/, accessed on 20 November 2025) [22] with default parameters, and the resulting alignments were manually refined in MEGA v7.

Phylogenetic datasets were constructed and analyzed separately for *Fusarium* and *Alternaria* isolates. The three gene regions (ITS, TEF1-α, and RPB2) were concatenated into a single partitioned dataset. The best-fit nucleotide substitution model was determined using ModelFinder v2.4.0 [23] based on the Bayesian Information Criterion (BIC). Bayesian inference (BI) phylogenetic analyses were conducted using MrBayes v3.2.6 [24] under the selected substitution model, with two independent parallel runs of 5 × 10^6^ generations. Trees were sampled every 1000 generations, and the first 25% of the sampled trees were discarded as burn-in. Posterior probability values were estimated from the remaining trees. Resulting trees were visualized and annotated with iTOL v5 [25].

### 2.5. Pathogenicity Assay

Pathogenicity tests were performed on asymptomatic 1–2-year-old branches (5–8 mm in diameter) attached to living fig trees (cv. ‘Zao-huang’) under natural field conditions in a commercial fig orchard in the Kashi region. Branch surfaces were surface-sterilized with 70% (*v*/*v*) ethanol, and a 4 mm-diameter cork borer was used to remove a bark disk 3–4 cm below a node. Mycelial plugs (4 mm in diameter) obtained from 5-day-old PDA cultures of each isolate were inserted into the wounds, while sterile PDA plugs were used as negative controls. The inoculation sites were covered with sterile, moistened cotton and sealed with Parafilm (Thermo Fisher Scientific, Waltham, MA, USA) to maintain high humidity. After 48 h, the Parafilm was removed, and inoculated branches were then allowed to continue growing on trees under natural conditions for 5 further days (7 days in total). Lesion development and expansion were monitored at regular intervals; during the experiment, the mean daily orchard temperature was ~26 °C, and relative humidity averaged ~60%. Each isolate was inoculated onto six branches (one wound per branch, spacing ≥ 10 cm), and the experiment was conducted three times independently. Each replication used branches from different trees located at separate positions within the orchard. To fulfill Koch’s postulates, fungi were re-isolated from the advancing margins of lesions, recultured, and molecularly identified to confirm their identity with the original inoculated isolates. Molecular identification of re-isolated fungi was performed using the same protocols as those used for initial fungal species identification.

### 2.6. Data Analysis

Lesion length data are presented as mean ± standard deviation (SD). Differences among treatments were analyzed using one-way analysis of variance (ANOVA), followed by Tukey’s honestly significant difference (HSD) post hoc tests (α = 0.05) using SPSS Statistics v26.0 software (IBM Corp., Armonk, NY, USA).

## 3. Results

### 3.1. Field Survey

**Figure 1:**
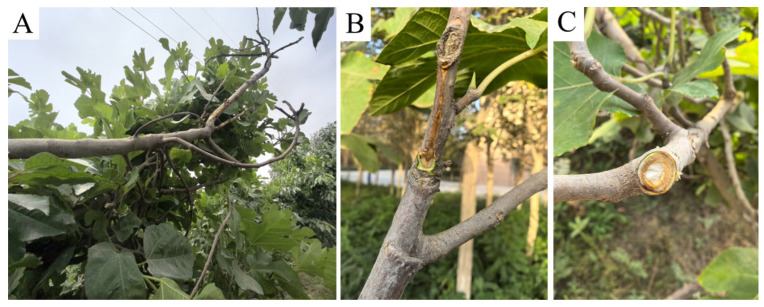
Canker and shoot dieback symptoms on fig trees: (**A**) typical canker lesions on a fig branch (bark dark-brown and sunken at the margin), (**B**) discolored brown xylem beneath the lesion, and (**C**) cross-section of an infected twig showing a sharp boundary between healthy and diseased tissues.

Fig branch canker predominantly affected trees older than 10 years, and lesions occurred most frequently on the trunk and primary scaffold branches (Figure 1A). Typical symptoms included elongated, dark-brown necrotic lesions on the bark. These lesions were distinctly sunken at the margins, forming a sharp boundary between healthy and diseased tissues (Figure 1C). Localized bark shrinkage and cracking were commonly observed, and transverse sections revealed a clear demarcation between infected and healthy wood tissues (Figure 1C). The cambial and phloem tissues beneath the bark were discolored brown, and browning of the xylem was also pronounced (Figure 1B). As lesions progressed, portions of the bark sloughed off or collapsed, resulting in wilting and subsequent death of leaves and shoots above the infected sites. Under severe disease pressure, entire branches and in some cases whole trees died.

### 3.2. Molecular Identification and Phylogenetic Analysis

**Figure 2:**
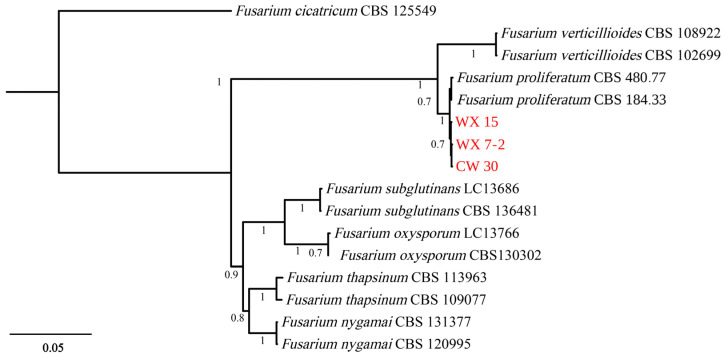
Bayesian phylogenetic tree inferred from the concatenated ITS, TEF-1α, and RPB2 sequence dataset. *Fusarium cicatricum* CBS 125549 was used as the out-group. Numbers at nodes are Bayesian posterior probabilities; only values ≥ 0.70 are shown. Isolates obtained in this study (*F. proliferatum* WX 15, WX 7-2, CW 30) are identified in red text.

Three *Fusarium* isolates, designated WX 15, WX 7-2, and CW 30, were obtained from fig branches exhibiting dieback symptoms. BLASTn analyses indicated that their ITS, TEF1-α, and RPB2 sequences shared ≥99% identity with published accessions of *F. proliferatum*, which preliminarily assigned these isolates to the *F. proliferatum* species complex. To further confirm their species-level identity, a multilocus phylogenetic analysis was performed. This analysis included the three newly obtained isolates, 13 reference strains representing closely related *Fusarium* species, and *Fusarium cicatricum* CBS 125549 as the outgroup. The concatenated dataset comprised 1582 nucleotide characters, including 517 bp from ITS, 215 bp from TEF1-α, and 850 bp from RPB2. Bayesian inference analysis showed that the three fig-derived isolates clustered in a strongly supported clade with a posterior probability of 1.00 together with the ex-type strain *F. proliferatum* CBS 184.33, thereby unambiguously confirming their identification as *F. proliferatum* (Figure 2).

**Figure 3:**
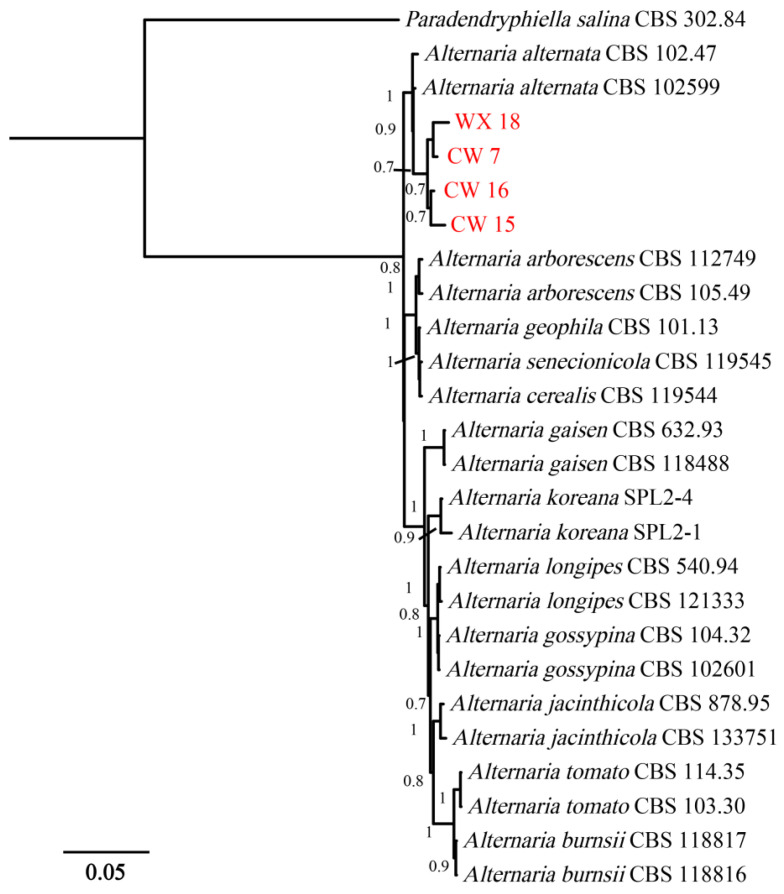
Bayesian phylogenetic tree inferred from the concatenated ITS, TEF-1α, and RPB2 sequence dataset. *Paradendryphiella salina* CBS 302.84 was used as the out-group. Numbers at nodes are Bayesian posterior probabilities; only values ≥ 0.70 are shown. Isolates obtained in this study (*A. alternata* WX 18, CW 7, CW 15, CW 16) are identified in red text.

Four *Alternaria* strains (WX 18, CW 7, CW 16, and CW 15) were also isolated from symptomatic fig branches. BLASTn queries revealed their ITS, TEF1-α, and RPB2 sequences shared ≥99% identity with reference accessions of *A. alternata*, preliminarily assigning these isolates to the *A. alternata* species complex. To confirm this taxonomic placement, a multilocus phylogenetic analysis was conducted. The analysis included the four newly obtained isolates, 22 reference strains representing closely related *Alternaria* species, and *Paradendryphiella salina* CBS 302.84 as the outgroup. The concatenated alignment comprised 1582 nucleotide positions, including 517 bp from ITS, 215 bp from TEF1-α, and 850 bp from RPB2. Bayesian inference analysis revealed that the four fig-derived isolates clustered in a strongly supported clade (posterior probability = 1.00) together with the ex-type strain *A. alternata* CBS 102599, unequivocally confirming their identification as *A. alternata* (Figure 3).

### 3.3. Morphological Characteristics

**Figure 4:**
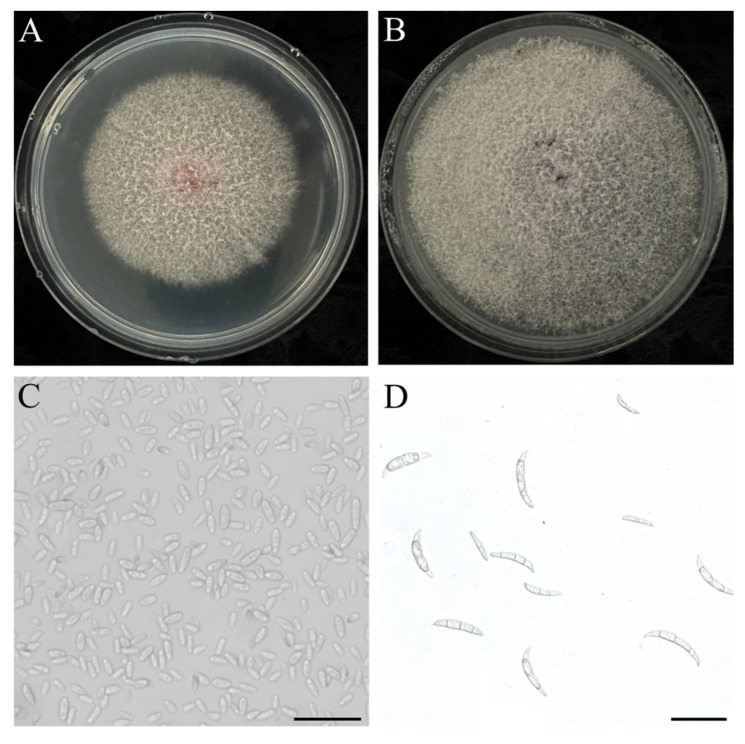
Colony morphology of *F. proliferatum* isolate WX 15 on PDA. Front view of (**A**) 5- and (**B**) 10 d-old colonies, (**C**) microconidia (scale bar = 50 µm), and (**D**) macroconidia (scale bar = 50 µm).

After culture on PDA at 25 °C in the dark for 5–10 d, colony morphology of representative isolates was described. The representative isolate *F. proliferatum* WX 15 produced floccose-to-cottony white-to-pale-pink aerial mycelia (Figure 4A,B). Hyphae were smooth, hyaline, branched, and septate. Microconidia were oval to obovoid, aseptate, or ellipsoid to clavate, with an average size of 8.42 × 2.51 µm (Figure 4C). Macroconidia were fusiform to falcate, with foot-shaped basal cells and elongated apical cells, bearing 3–5 septa, and measuring 15.21 ± 7.8 × 2.52 ± 1.1 µm on average (Figure 4D).

**Figure 5:**
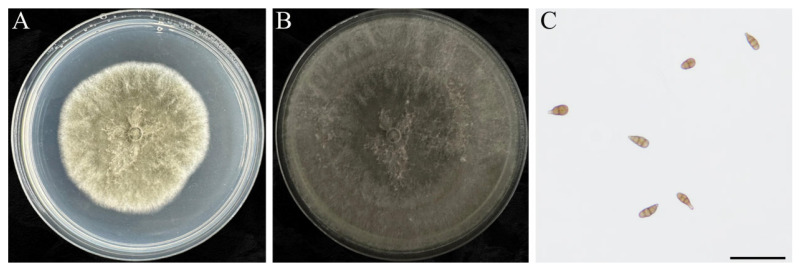
Colony morphology of *A. alternata* isolate CW 7 on PDA. Front view of (**A**) 5- and (**B**) 10 d-old colonies, and (**C**) conidia (scale bar = 50 µm).

Colonies of *A. alternata* isolate CW 7 were initially white to gray-white, gradually turning brown; mycelia were sparse and denser centrally (Figure 5A,B). Conidia were obclavate to pyriform, ranging 22–43 µm × 8–14 µm in size, with a long beak equal to or slightly longer than the conidial body, and usually with 3–5 transverse septa. These characteristics are consistent with those of *A. alternata* (Figure 5C).

### 3.4. Pathogenicity Verification Test

**Figure 6:**
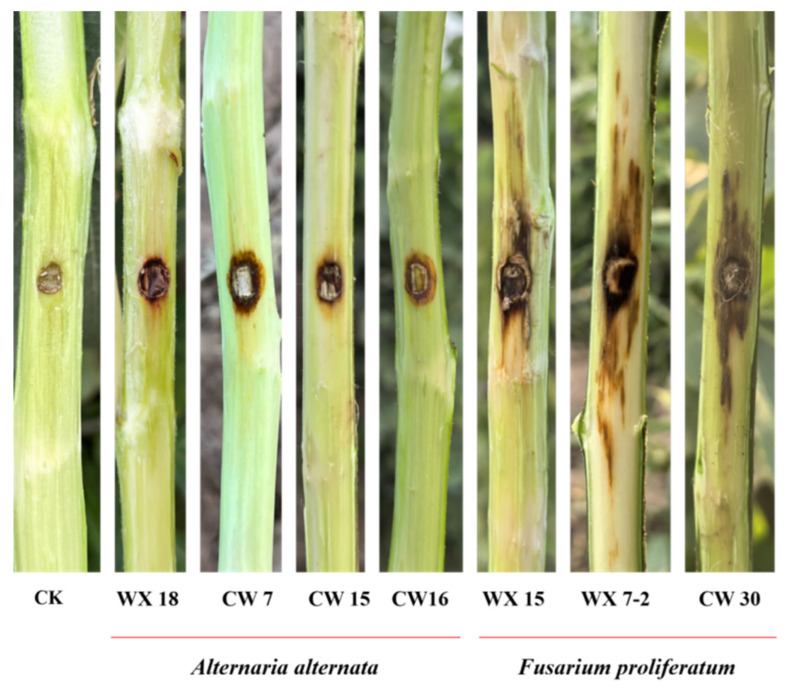
Symptom development on living fig branches 7 d post inoculation under field conditions. CK, non-inoculated control; WX 18, CW 7, and CW 15, *A. alternata* isolates; CW 16, WX 15, WX 7-2, and CW 30, *F. proliferatum* isolates. All inoculated branches developed dark-brown canker lesions on intact branches, with clear differences in lesion severity among isolates.

**Figure 7:**
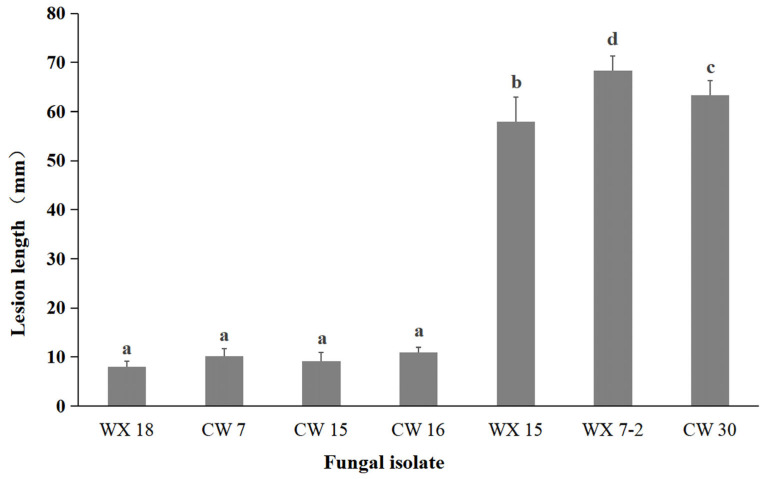
Comparison of mean lesion length (mm) induced by different fungal species and strains on living fig branches in pathogenicity assays. Bars represent the mean lesion length of six independently inoculated branches per isolate; error bars indicate the standard error of the mean (SE). Different letters above bars denote statistically significant differences among treatments based on one-way ANOVA followed by Tukey’s HSD test at *p* = 0.05.

To evaluate the pathogenicity of isolated fungi on fig branches, seven representative isolates—three *A. alternata* isolates (WX 18, CW 7, and CW 15) and four *F. proliferatum* isolates (CW 16, WX 15, WX 7-2, and CW 30)—were inoculated onto 1–2-year-old branches attached to living fig trees. At 3 days post-inoculation (dpi), slight sunken discoloration of the bark was observed around the inoculation sites. By 7 dpi, all inoculated branches developed dark-brown canker-like lesions that closely resembled the symptoms observed under natural field conditions, whereas control branches remained symptomless; the inoculated branches were only detached from the host trees after symptom evaluation for high-resolution image acquisition of lesion phenotypes (Figure 6). Mean lesion lengths differed significantly among fungal isolates. Lesions caused by *A. alternata* isolates (~7 mm) were significantly shorter than those induced by *F. proliferatum* isolates (13–18 mm) (Figure 7). To fulfill Koch’s postulates, fungi were re-isolated from the advancing margins of representative lesions formed on inoculated living branches. Subsequent morphological examination and molecular identification confirmed that the recovered isolates were identical to the original inoculum.

## 4. Discussion

Through systematic field surveys, pathogen isolation, morphological characterization, multi-locus phylogenetic analyses, and artificial inoculation, we demonstrate that *A. alternata* and *F. proliferatum* are the primary causal agents of fig branch canker in southern Xinjiang. Both fungi consistently infect lignified fig branches and produce lesions identical to those observed in the field. Both were also successfully re-isolated from diseased tissue, fulfilling Koch’s postulates. These results conclusively reveal the pathogen complex of fig branch canker in this region. Neither *A. alternata* nor *F. proliferatum* has previously been reported as a causal agent of fig branch canker. Their consistent isolation from symptomatic tissues in southern Xinjiang, together with pathogenicity confirmation in the present study, extends the known pathogen spectrum and highlights pronounced regional variability in the etiology of fig branch diseases.

To date, fig branch canker and dieback have been predominantly associated with fungal species belonging to the families Botryosphaeriaceae and Diaporthaceae, with *Phomopsis cinerascens*, *Lasiodiplodia theobromae*, and *Neofusicoccum parvum* being the most frequently reported pathogens in Iran, Turkey, and Italy [3,4,5,6]. More recently, *Neocosmospora* species have been identified as emerging agents of fig branch necrosis in the Mediterranean region and Iran [8], further expanding the known disease complex.

*Fusarium proliferatum* has been reported to infect branches or trunks of several woody hosts and to cause canker diseases in date palm, almond, and pistachio [12,26,27]. These reports support its ability to colonize xylem tissues and induce tissue necrosis. This capacity is consistent with the pronounced canker symptoms observed on fig branches in our inoculation assays. In contrast, *A. alternata* has long been regarded as a major pathogen causing leaf spot and fruit rot in numerous crops [13]. Recent studies have shown that it can also induce typical canker and dieback symptoms on apple branches [15], and it is widely recognized as an endophytic or opportunistic pathogen [28]. Our results demonstrate that *A. alternata* can independently infect fig branches and induce stable canker symptoms, rather than acting solely as a secondary saprophyte or incidental associate.

Recent studies have increasingly emphasized the role of the pathobiome in woody plant health, suggesting that interactions among multiple fungi often exacerbate canker development [29,30]. Our finding that *A. alternata* and *F. proliferatum* coexist as primary pathogens is consistent with this perspective. In similar arid environments, Fusarium species have been reported to exploit host stress caused by temperature fluctuations. A comparable pattern has been observed in recent outbreaks of stone fruit canker in Shaanxi [31]. When considered within this broader context, the etiology of fig branch canker in southern Xinjiang appears to be shaped by both regional climatic conditions and the evolving pathogenic behavior of these widespread fungi.

In summary, this study demonstrates that *A. alternata* and *F. proliferatum* are causal agents of fig branch canker in southern Xinjiang, thereby enriching the regional pathogen composition associated with this disease. These findings provide essential baseline data for future etiological research and for the development of effective management strategies targeting fig branch diseases.

## 5. Conclusions

We identified and characterized the primary fungal pathogens responsible for fig branch canker in the Kashi region of Xinjiang, revealing previously unreported causal agents and their diversity. This work provides a scientific foundation for establishing region-specific disease monitoring and sustainable management systems. Future studies should focus on elucidating infection mechanisms, mycotoxin production, and the evolution of fungicide resistance in these pathogens. In addition, efforts are needed to screen highly effective and low-toxicity control compounds and to identify resistant fig cultivars, thereby supporting the sustainable development of the fig industry in Xinjiang.

## Data Availability

The original contributions presented in this study are included in the article. Further inquiries can be directed to the corresponding authors.

## References

[1] Nelson A.H., Hudler G.W. (2007). A Summary of North American Hardwood Tree Diseases with Bleeding Canker Symptoms. Arboric. Urban For. (AUF).

[2] Gauthier N., Kaiser C., Owen W. (2012). Woody Plant Disease Management Guide for Nurseries & Landscapes.

[3] Çeliker N.M., Michailides T.J. (2012). First report of *Lasiodiplodia theobromae* causing canker and shoot blight of fig in Turkey. New Dis. Rep..

[4] Banihashemi Z., Javadi A.R. (2009). Further investigations on the biology of *Phomopsis cinerascens*, the cause of fig canker in Iran. Phytopathol. Mediterr..

[5] Gusella G., Gugliuzzo A., Guarnaccia V., Martino I., Aiello D., Costanzo M.B., Russo A., Groenewald J.Z., Crous P.W., Polizzi G. (2024). Fungal species causing canker and wilt of Ficus carica and evidence of their association by bark beetles in Italy. Plant Dis..

[6] Aiello D., Gusella G., Fiorenza A., Guarnaccia V., Polizzi G. (2020). Identification of *Neofusicoccum parvum* causing canker and twig blight on *Ficus carica* in Italy. Phytopathol. Mediterr..

[7] Gusella G., Fiore G., Vitale A., Felts D.G., Michailides T.J. (2023). New findings on the effects of different factors involved in fig limb dieback caused by *Neoscytalidium dimidiatum* in California. Eur. J. Plant Pathol..

[8] Bolboli Z., Mostowfizadeh-Ghalamfarsa R., Sandoval-Denis M., Jafari M., Crous P.W. (2022). *Neocosmospora caricae* sp. nov. and *N. metavorans*, two new stem and trunk canker pathogens on Ficus carica in Iran. Mycol. Prog..

[9] Basavand E., Khodaygan P., Rahimian H., Doonan J.M., Pakdin-Parizi A. (2021). First report of bacterial canker of fig trees caused by *Brenneria nigrifluens*. J. Phytopathol..

[10] Park K.-T., Ten L., Hong S.-M., Nam S.-W., Back C.-G., Lee S.-Y., Jung H.-Y. (2024). First Report of *Pectobacterium aroidearum* Causing Soft Rot on Ficus carica in Korea. Res. Plant Dis..

[11] Zakaria L. (2023). *Fusarium* species associated with diseases of major tropical fruit crops. Horticulturae.

[12] López-Moral A., Antón-Domínguez B.I., Lovera M., Arquero O., Trapero A., Agustí-Brisach C. (2024). Identification and pathogenicity of Fusarium species associated with wilting and crown rot in almond (*Prunus dulcis*). Sci. Rep..

[13] Rotem J. (1994). The Genus Alternaria: Biology, Epidemiology, and Pathogenicity.

[14] Zhang M.-J., Zheng X.-R., Li H., Chen F.-M. (2023). *Alternaria alternata*, the Causal Agent of a New Needle Blight Disease on Pinus bungeana. J. Fungi.

[15] Li Z., Li H., Zhang J., Zhang S., Zhao Q., Cheng C., Zhang Y. (2024). Characterization of Fungal Species Isolated from Cankered Apple Barks Demonstrates the *Alternaria alternata* Causing Apple Canker Disease. J. Fungi.

[16] Slippers B., Wingfield M.J. (2007). *Botryosphaeriaceae* as endophytes and latent pathogens of woody plants: Diversity, ecology and impact. Fungal Biol. Rev..

[17] Sun H., Yali W., Wang W., Zupaila N., Wang L., Ma R. (2025). Study on Influencing Factors and Low-Temperature Treatment of Walnut Canker Disease in Hotan County, Xinjiang. Forests.

[18] Kranjec Orlović J., Diminić D., Ištok I., Volenec I., Hodak L., Grubešić M., Tomljanović K. (2024). Fungal Presence and Changes of Wood Structure in Bark Stripping Wounds Made by Red Deer (*Cervus elaphus* L.) on Stems of Fraxinus angustifolia (Vahl). Forests.

[19] Luo Y., Lichtemberg P.S.F., Niederholzer F.J.A., Lightle D.M., Felts D.G., Michailides T.J. (2019). Understanding the Process of Latent Infection of Canker-Causing Pathogens in Stone Fruit and Nut Crops in California. Plant Dis..

[20] Fang Z.D. (1998). Plant Disease Research Methods.

[21] He J., Li D.W., Cui W.L., Huang L. (2024). Seven new species of *Alternaria* (Pleosporales, Pleosporaceae) associated with Chinese fir, based on morphological and molecular evidence. MycoKeys.

[22] Katoh K., Rozewicki J., Yamada K.D. (2019). MAFFT online service: Multiple sequence alignment, interactive sequence choice and visualization. Brief. Bioinform..

[23] Kalyaanamoorthy S., Minh B.Q., Wong T.K., Von Haeseler A., Jermiin L.S. (2017). ModelFinder: Fast model selection for accurate phylogenetic estimates. Nat. Methods.

[24] Ronquist F., Teslenko M., Van Der Mark P., Ayres D.L., Darling A., Höhna S., Larget B., Liu L., Suchard M.A., Huelsenbeck J.P. (2012). MrBayes 3.2: Efficient Bayesian phylogenetic inference and model choice across a large model space. Syst. Biol..

[25] Letunic I., Bork P. (2021). Interactive Tree Of Life (iTOL) v5: An online tool for phylogenetic tree display and annotation. Nucleic Acids Res..

[26] Abdalla M.Y., Al-Rokibah A., Moretti A., Mulè G. (2000). Pathogenicity of Toxigenic *Fusarium proliferatum* from Date Palm in Saudi Arabia. Plant Dis..

[27] Ören E., Palacıoğlu G., Bayraktar H. (2023). First report of *Fusarium proliferatum* causing canker on apricot (*Prunus armeniaca*) in Turkey. J. Plant Pathol..

[28] DeMers M. (2022). Alternaria alternata as endophyte and pathogen. Microbiology (Reading).

[29] Habib W., Carlucci M., Cavalieri V., Carbotti C., Nigro F. (2025). Unveiling a Disease Complex Threatening Fig (*Ficus carica* L.) Cultivation in Southern Italy. Plants.

[30] Monod V., Hofstetter V., Viret O., Zufferey V., Gindro K., Croll D. (2025). Landscape-scale endophytic community analyses in replicated grapevine stands reveal that dieback disease is unlikely to be caused by specific fungal communities. Appl. Environ. Microbiol..

[31] Ding D., Shao Y., Zhao J., Lin J., Zhang X., Wang X., Xu X., Xu C. (2024). Identification and pathogenicity of *Alternaria* and *Fusarium* species associated with bagged apple black spot disease in Shaanxi, China. Front. Microbiol..

